# Mechanism and enhancement of lipid accumulation in filamentous oleaginous microalgae *Tribonema minus* under heterotrophic condition

**DOI:** 10.1186/s13068-018-1329-z

**Published:** 2018-12-11

**Authors:** Hui Wang, Yan Zhang, Wenjun Zhou, Leksawasdi Noppol, Tianzhong Liu

**Affiliations:** 1 Key Laboratory of Biofuels, Qingdao Institute of Bioenergy and Bioprocess Technology, Chinese Academy of Sciences, Qingdao, 266101 Shandong People’s Republic of China; 2 University of Chinese Academy of Sciences, Beijing, 100049 People’s Republic of China; 3 Bioprocess Research Cluster, School of Agro-Industry, Faculty of Agro-Industry, Chiang Mai University, Chiang Mai, 50200 Thailand

**Keywords:** *Tribonema minus*, Lipids, Heterotrophic, Transcriptomics, Metabolomics

## Abstract

**Background:**

The filamentous microalgae *Tribonema minus* accumulates large amounts of lipids under photoautotrophic condition, while under heterotrophic condition, the lipid content decreased dramatically. Determination of the differences in metabolic pathways between photoautotrophic and heterotrophic growth will provide targets and strategies for improvement of lipid accumulation in heterotrophic cells.

**Methods:**

The metabolic differences between photoautotrophically and heterotrophically cultivated *T. minus* cells were studied by comparing the growth, biochemical compositions and transcriptomic and metabolomic profiles of the cells. Based on comparative transcriptomic and metabolomic studies, we generated a global model of the changes in central carbon metabolism and lipid biosynthetic pathways that occur under photoautotrophic and heterotrophic growth conditions. Moreover, the specific effects of supplementation with exogenous key metabolic intermediates on the lipid accumulation in heterotrophic culture were analyzed.

**Results:**

Compared to photoautotrophic cultures, heterotrophic cultures exhibited enhanced biomass levels and carbohydrate content, but decreased lipid accumulation. These effects were accompanied by low expression levels of genes involved in glycolysis, de novo fatty acids biosynthesis and lipid biosynthesis, and high levels of genes involved in gluconeogenesis. In addition, the levels of key metabolites involved in glycolysis/gluconeogenesis were elevated in abundance, whereas those of certain fatty acids and citric acid were decreased in heterotrophic cultures. Upon supplementation with exogenous potassium palmitate, the lipid content increased dramatically in heterotrophically cultivated *T. minus*.

**Conclusion:**

An insufficient supply of carbon precursors caused the low levels of lipid accumulation during heterotrophic cultivation. Appropriate carbon metabolite supplementation based on the metabolomic data was shown to promote lipid accumulation. Moreover, gene regulatory metabolic targets were also identified via omics analysis.

## Introduction

Continuous increasing pressures of fuel production and environmental protection have led to surge in the search for alternative and renewable energy [1]. Microalgae are considered to be promising candidates for liquid biofuel production because these organisms have high photosynthetic efficiencies, growth rate, and biological yield, and microalgal cultivation does not compete with food production for arable land [2, 3]. Some microalgal strains produce lipids for energy storage, and these lipids are stored intracellularly as reserve material during the vegetative period of growth [4]. Among these microalgae, the yellow–green microalga *Tribonema* sp., which can accumulate large amounts of lipid droplets in the cytoplasm, is the first reported filamentous oleaginous microalgae. Due to its filamentous character, this species has advantages over other oleaginous microalgae for cultivation adaptability and harvesting [5].

In our previous research, we found that *Tribonema* sp. can accumulate lipids up to 62% of the dry cell weight under photoautotrophic condition [6]. Moreover, *Tribonema* sp. is also capable of using glucose as a carbon source for rapid heterotrophic growth, further demonstrating that this specie is among the best oil feedstock microalgae for biodiesel production [7]. However, the heterotrophic mode promotes the biomass accumulation of *Tribonema* sp. but sharply decreases the lipid content [8]. Therefore, it is quite necessary to determine the mechanism underlying lipid accumulation and develop an effective strategy for enhancing lipid accumulation without reducing the biomass during heterotrophic fermentation.

To date, transcriptomic studies that have been used to investigate changes in the responses to environmental perturbations are relatively comprehensive, and the identification of the intrinsic mechanism that controls lipid biosynthesis in oleaginous species has already been performed in model oleaginous microalgae [9, 10]. Microarray-based transcriptomics and RT-PCR have provided useful information regarding the cellular response to environmental changes at gene expression level [11, 12], but the information does not necessarily reflect the changes in metabolite activity [13]. As a method to study and measure the levels of the small-molecule metabolites that constitute biochemical networks, metabolomics has recently been applied to studies of various microalgae and is considered as a valuable tool for the analysis of cellular metabolism [14–16].

In addition to optimizing cultivation conditions, an alternate approach to the modulation of lipid pathways was proposed, namely, the identification of chemical triggers that can directly elicit the lipid accumulation [17, 18]. In recent studies, some cell metabolite markers were used to enhance lipid accumulation, which might represent a practical approach to achieve high levels of lipid accumulation in algae [19]. Moreover, Cheng et al. used metabolomic analysis to identify potential markers for improving lipid accumulation of microalgae [20]. However, thus far, no similar investigation has been conducted to identify chemical triggers for enhancement of lipid accumulation in *Tribonema* sp., especially in heterotrophic cultivation mode.

The present work focused on the mechanism and regulation of lipid accumulation in *Tribonema* cells during heterotrophic cultivation. The aims of this study were (1) to test the changes in cell ultrastructure, physiology and biochemistry of *Tribonema* sp. under photoautotrophic and heterotrophic cultivation; (2) to elucidate the complex metabolic networks associated with lipid accumulation in microalgal cells via de novo transcriptomics in combination with metabolomic analysis; and (3) to evaluate the effects of potential metabolite markers on the enhancement of lipid accumulation under heterotrophic growth.

## Materials and methods

### Microalgae and culture conditions

The yellow–green microalgae *Tribonema minus* SAG 880-3 was obtained from the Culture Collection of Algae of Gottingen University and maintained in our laboratory. The cells initially were maintained and cultivated in BG11 medium at 23 ± 1 °C. For photoautotrophic cultivation, microalgal cells in the logarithmic growth phase were inoculated into 800-mL column photobioreactors containing 600 mL of fresh medium with continuous light at a light density of 100 μmol/m^2^s and bubbled with compressed air containing 1.5% CO_2_. The carbon source was supplied with glucose at an initial concentration of 20 g/L, while nitrogen was pumped into mix the culture, and aluminum foil was used to wrap the column photobioreactors for dark treatment for heterotrophic cultivation. Each experiment was performed in three times, and biomass concentration was determined by measured dry weight [21] of cells everyday.

### Determination of biochemical composition

#### Chlorophyll and total carotenoid content

Chlorophyll content was determined by following the methods described by Work et al. [22]. Briefly, aliquots of microalgal broth under photoautotrophic and heterotrophic conditions were harvested, and the cell pellet was re-suspended in 95% (v/v) ethanol. The test tubes were maintained overnight in the dark at 4 °C for pigment extraction. Cellular debris was removed by centrifugation at 4000×*g* for 10 min, and the optical densities of the supernatant at 665, 649 and 470 nm were determined using a UV–vis spectrophotometer. The chlorophyll a, chlorophyll b and total carotenoid levels were calculated as described previously [23].

#### Lipid accumulation analysis

Lipid extraction was performed as described previously with slight modifications [24]. Briefly, 50 mg of lyophilized cell powder was mixed with 7.5 mL of chloroform:methanol (2:1) solvent with shaking overnight. The mixture was centrifuged at 8000×*g* for 10 min and the supernatant was collected, and the residual biomass was extracted once more. All the supernatants were combined, and chloroform and 1% sodium chloride solution were added to a final volumetric ratio of 1:1:0.9 (chloroform:methanol:water). After centrifugation, the chloroform phase was carefully transferred to a vial and dried to constant weight at 60 °C under nitrogen flow. Finally, the total lipids were obtained, and the total lipid content was calculated as a percentage of the dry weight of microalgae.

#### Protein and carbohydrate assessment

Protein extraction was performed according to methods described by Turgay et al. [25]. Freeze-dried cell pellets were re-suspended in lysis buffer (50 mM Tris–HCl pH 8.0, 2% SDS, 10 mM EDTA, protease inhibitor mix), subjected to sonication for 1 min at 60% powder, frozen in liquid nitrogen for 1 min, thawed, and centrifuged at 10,000×*g* for 20 min at 4 °C. The supernatant was used for determination of protein concentration with Bradford method [26]. The carbohydrate content was determined using the modified quantitative scarification (QS) method reported by the National Renewable Energy Laboratory (NREL), USA [27].

### Transcriptomic analysis

#### De novo transcriptome assembly

Fresh microalgal cells grown in photoautotrophic and heterotrophic modes were harvested during the stationary growth phase. Total RNA of algal cells was extracted from ground frozen pellets using the TransZol Plant RNA Kit (Transgen, Beijing, China). Paired-end cDNA sequencing libraries were prepared using IlluminaTruSeq Stranded Total RNA Library Preparation Kit according to the manufacturer’s protocol. The cDNA libraries were sequenced with the Illumina HiSeq 2000 platform (Shanghai Personal Biotechnology Co., Ltd).

#### RNA-Seq data analysis

The raw reads were firstly assessed to obtain high-quality Illumina sequencing data with clean reads, and then processed to remove Illumina adapters and low-quality reads as described in previous study [28]. The assembled genes were annotated by BLASTX searches against the databases, mainly NR protein database and COG database. Additional annotations were obtained from the Kyoto Encyclopedia of Genes and Genomes (KEGG) gene and protein family database through the KEGG Automatic Annotation Server (KAAS) [29]. Genes with differential expression between photoautotrophic and heterotrophic modes were identified using the number of mapped reads as EdgeR inputs (http://www.bioconductor.org/packages/release/bioc/html/degeR.html). Genes were defined as being differentially expressed, if they exhibited a change of twofold or more between two samples and a false discovery rate (FDR) of 5% or less.

### Metabolomic analysis

#### Metabolomic extraction

Metabolomic extraction was conducted as described previously with some modification [30, 31]. Microalgal cells were collected by filtration of 150 mL of culture through a pre-washed Whatman GF/F filter under reduced pressure (800 mbar). Then, the cells were immediately frozen in liquid nitrogen and then stored at − 80 °C before further use. Lyophilized cells were ground by a grinder (MM 400, Retsch) for approximately 1 min at 4 °C, and 100 mg of powder was re-suspended in 1 mL of cold 70% cold methanol and frozen in liquid nitrogen overnight and thawed three times. Supernatants were collected by centrifugation at 10,000×*g* for 10 min at 4 °C, and the samples were further filtrated with 0.22-µm microporous filters and concentrated in vials for LC–MS analysis.

#### LC–MS examination

Metabolomics analysis was performed on a HPLC system (Agilent Technologies, Waldbronn, Germany) coupled to an Agilent 6410 triple quadrupole mass analyzer equipped with an electronic spray ionization (ESI) source [32]. A Synergi Hydro RP (C18, 100 mm × 2.1 mm, 4 μm 80Α particles; Phenomenex, Torrance, CA) column was used for the metabolite separation. Mobile phase A (MPA) was an aqueous 10 mM tributylamine solution adjusted to pH 4.95 with acetic acid, and mobile phase B (MPB) was 100% HPLC-grade methanol. Constant flow rate of 0.2 mL/min was maintained. Data processing and statistical analysis were performed using Agilent Mass Hunter workstation LC/QQQ acquisition software (version B.04.01) and analyzed using Agilent Mass Hunter workstation qualitative analysis software (version B.04.00). MS was performed in negative mode for multiple reaction monitoring (MRM) development, method optimization, and sample analysis. The injection volume for all samples was 10 μL, capillary voltage was 4000 V, and the nebulizer gas flow rate and pressure were 10 L/min and 50 psi, respectively. The nitrogen nebulizer gas temperature was 300 °C.

#### Metabolomics analysis

Metabolomic data were normalized to an internal control and cell number, and then subjected to principal component analysis (PCA) using SIMCA-P 11.5 [33]. Additionally, to determine the pattern of metabolic regulation, the differential metabolites were identified as responsive biomarkers. These metabolites were selected based on a combination of a statistically significant threshold of variable influence on projection (VIP) values obtained from the orthogonal partial least squares discriminant analysis (OPLS-DA) model and *p* values from a two-tailed Student’s *t*-test on the normalized peak areas. The names of the metabolites and the times of change were input into the Omics Bean software (Gene for Health Biological Technology Co., Shanghai, China) for bioinformatic analysis of metabolic pathways. The metabolic information was mapped to the KEGG database to identify enriched KEGG pathways.

### Integrated lipid biosynthesis-associated changes in the transcriptome and metabolome under heterotrophic condition

By employing the transcriptomic and metabolomic data sets acquired as detailed above, integrated biochemical maps were constructed to illustrate the global changes in primary metabolism that occur with heterotrophic cultivation. The changes in carbon precursor flow scheme that leads to lipid biosynthesis were further analyzed. The basic framework was constructed based on KEGG pathways.

### Effect of potential marker supplementation on growth and lipid accumulation during heterotrophic cultivation

Based on the acquired transcriptomic and metabolomic data, six potential metabolite markers (mannitol, dulcitol, *N*-methyltyramine, citrate, succinate and potassium palmitate) were chosen and examined for their effects on the lipid accumulation in *T. minus* under heterotrophic condition. Discrete concentrations of mannitol (0.5, 1, 1.5 g/L), dulcitol (0.5, 1, 1.5 g/L), *N*-methyltyramine (50, 100, 200 mg/L) citrate (0.5, 1, 1.5 g/L), succinate (0.5, 1, 1.5 g/L) and potassium palmitate (0.5, 1, 2 g/L) were individually added into BG11 culture medium with glucose, and the microalgal cells were inoculated and cultured in 800-mL column bioreactors at 23 ± 1 °C in darkness. Each treatment was in performed in triplicate. The dry weights were analyzed every 2 days and the lipids were extracted and the contents were calculated at stationary phase.

## Results and discussion

### Comparison of the growth and biochemical compositions accumulation of *T. minus* under photoautotrophic and heterotrophic conditions

**Fig. 1:**
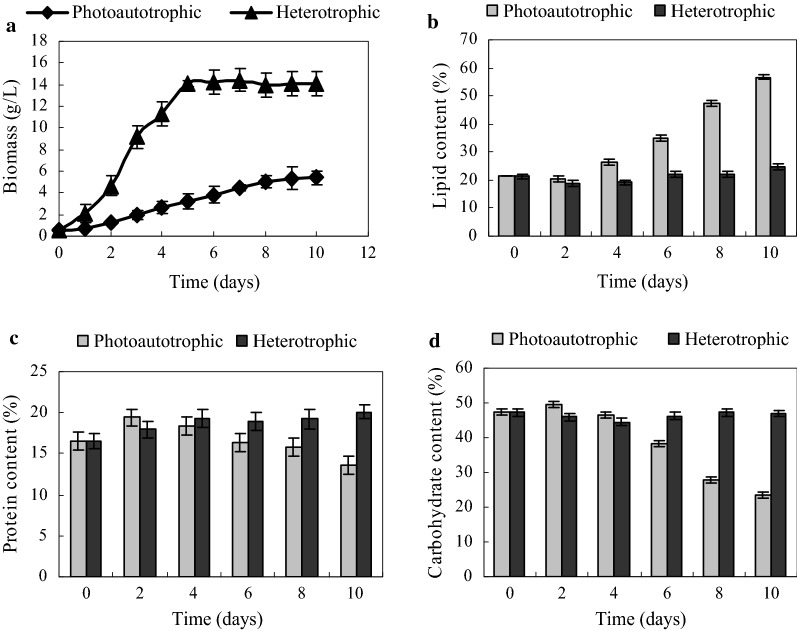
Growth and biochemical composition accumulation of *T. minus* under photoautotrophic and heterotrophic conditions. **a** Dry weight biomass of *T. minus* under different culture conditions; **b** lipid content of the dry weight in *T. minus* under different culture conditions; **c** protein content of the dry weight in *T. minus* under different culture conditions; **d** carbohydrate content of the dry weight in *T. minus* under different culture conditions

Microalga *Tribonema minus* cells were grown in column photobioreactors under photoautotrophic and heterotrophic conditions. Figure 1a shows that the cell growth differed from the two conditions: cells started to multiply at a rather high rate under heterotrophic conditions after inoculation, and the biomass reached the highest level of 14.3 g/L at day 5; however, cell growth under photoautotrophic condition exhibited a slow upward trend during the 10 days of cultivation. The stationary phase was reached by day 8 under photoautotrophic condition. The biomass concentration was only 5.36 g/L, which was significantly lower than that obtained under heterotrophic condition.

The lipid content was greater than 55% under photoautotrophic condition (Fig. 1b); however, the value was much lower under heterotrophic cultivation, with the highest of 24.72% observed at day 10. This finding is not consistent with the previous study conducted on a different green microalgae *Chlorella protothecoides*, where glucose was seen to trigger a high growth rate and lead to high levels of oil accumulation [34, 35]. However, this finding in *T. minus* is similar to the results observed for *Monoraphidium* sp. which exhibits increased biomass density but decreased the lipid content when switched organism from photoautotrophic to heterotrophic condition [36].

The protein content in *T. minus* under photoautotrophic condition first increased till day 2, and then presented a tendency of decrease till day 10 (Fig. 1c). By contrast, *T. minus* cells grown under heterotrophic condition exhibited a slow increase in protein content throughout the cultivation period. However, only slight changes in protein accumulation were observed between photoautotrophic and heterotrophic cultivation, in contrast to the significant changes observed in lipid content. Under heterotrophic condition, the carbohydrate content was 44.56–47.38% of the dry weight over 10 days of cultivation (Fig. 1d). However, the carbohydrate content under photoautotrophic condition decreased consistently, and the value was 50% of that obtained under heterotrophic condition by day 10.

In summary, this finding indicates that greatly decreased carbohydrate biosynthesis results in the repartitioning of carbon to lipids under photoautotrophic condition. Although the cytoplasm of *T. minus* contains no starch, this result is quite similar to that of a previous study of *Chlorella vulgaris* [37]. In addition, under heterotrophic condition, *T. minus* cells possess high levels of carbohydrate and low levels of lipid, which are in contrast to the levels observed during *Chlorella protothecoides* fermentation [38]. To further identify differences between photoautotrophic and heterotrophic lipid metabolism in *T. minus* and propose a targeted strategy to improve lipid accumulation under heterotrophic condition, quantitative proteomics and metabolite profiling of microalgal cells under heterotrophic condition were performed, and the results were compared with those of cells cultured under photoautotrophic condition.

### Alterations in pigment concentrations of *T. minus* under photoautotrophic and heterotrophic conditions

We also measured the pigment concentrations of *T. minus* cells at different growth phases to assess the impact of different culture modes. Based on the results presented in Table 1, chlorophyll *a* was the most common pigment found in both photoautotrophic and heterotrophic conditions, followed by total carotene and chlorophyll *b*. In the present study, although the chlorophyll *a* and *b* contents decreased under both conditions, there remained significant differences between photoautotrophic and heterotrophic conditions. Under heterotrophic condition, chlorophyll *a* was present at 1.87 μg/mg, and the value reduced by 69% during the stationary phase compared to the value under photoautotrophic condition. Meanwhile, the chlorophyll *b* content under heterotrophic condition reduced to 0.52 μg/mg, which was much lower than the value of 1.16 μg/mg observed under photoautotrophic condition. In conclusion, heterotrophic condition significantly decreased the chlorophyll content of *T. minus* compared to photoautotrophic condition, which is consistent with the results of a previous study on *Neochloris oleoabundans* [39]. In addition, the reduced chlorophyll contents under heterotrophic condition led to the yellow appearance of the culture broth, in contrast to cells growing under photoautotrophic cultivation, which remained green in color.

**Table 1:** Pigment levels in microalgae *T. minus* under photoautotrophic and heterotrophic conditions

Conditions	Growth phase	Chlorophyll*a* (μg/mg)	Chlorophyll*b* (μg/mg)	Total carotene (μg/mg)
Photoautotrophic	Initial	8.81 ± 0.11	1.86 ± 0.03	1.85 ± 0.08
	Logarithmic	9.86 ± 0.25	1.35 ± 0.02	3.17 ± 0.07
	Stationary	5.97 ± 0.14	1.16 ± 0.07	4.96 ± 0.21
Heterotrophic	Initial	8.81 ± 0.09	1.86 ± 0.04	1.85 ± 0.00
	Logarithmic	3.14 ± 0.02	0.73 ± 0.01	1.73 ± 0.04
	Stationary	1.87 ± 0.01	0.52 ± 0.00	1.52 ± 0.06

Results are presented as the mean ± standard deviations (*n* = 3)

The variation trends of total carotenoid accumulation under the two conditions were different. The carotenoid content in heterotrophic cells decreased slightly, which was in contrast to that in photoautotrophic cells, which increased from 1.85 to 4.96 µg/mg. Thus, we concluded that the chlorophyll *a* and *b* levels were lower and carotenoid content was higher under photoautotrophic condition than those under heterotrophic condition. Carotenoids from microalgae serve as photoprotective compounds that prevent photo-oxidative damage to photoautotrophic cells [40]. Thus, higher carotenoid levels indicate higher cell survival rates. Carotenoid production is always observed at the start of rapid lipid accumulation in microalgal cells. Previous research has shown that some carotenoids, such as β-carotene, accumulate in globules outside the chloroplast and protect the reaction center from excessive excitation by absorbing light and reducing the amount of energy transferred to the reaction center [41].

### De novo transcriptome assembly and annotation

**Fig. 2:**
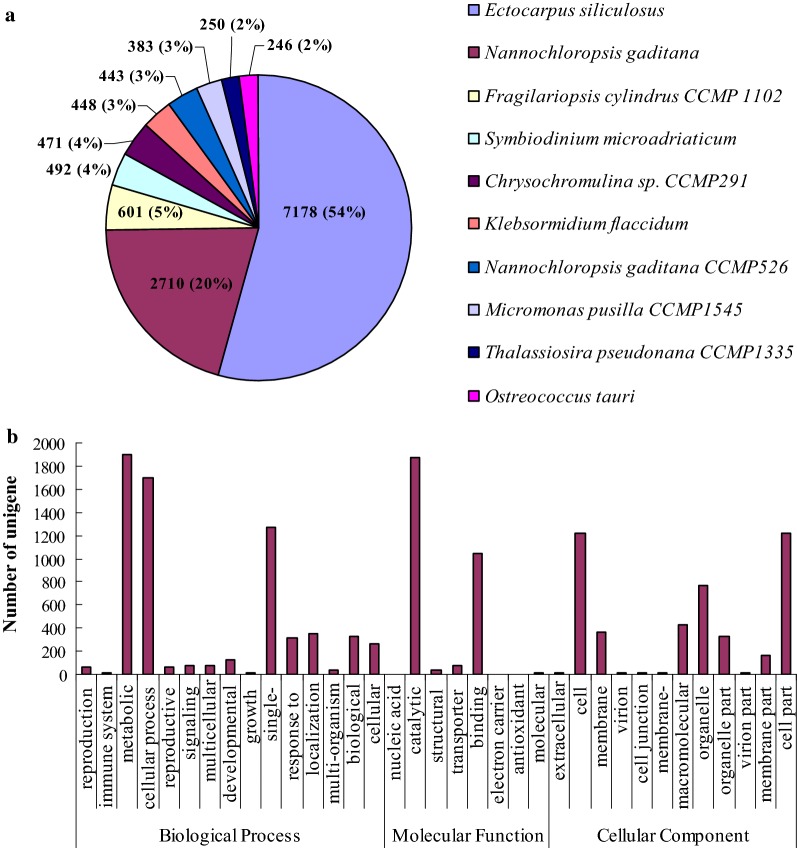
A top blast species distribution for BLASTx matches in *T. minus* and gene ontology (GO) assignment. **a** Distribution of BLAST top hit species; **b** Go categories in *T. minus*

To identify the transcriptomic changes that occur during lipid accumulation, RNA-seq analysis was performed using samples of photoautotrophic and heterotrophic cultures. The RNA-seq reads from six libraries (three replicates per condition) were combined and subjected to de novo transcriptome assembly by Trinity. The transcriptome assembly yielded 58,211 transcripts with the N50 of 1311 bp and an average transcript length of 896 bp. Subsequently, the assembled transcripts were annotated by BLASTX against the NR protein database. Of the 58,211 transcripts, 15,038 transcripts had at least one significant hit, as identified by the BLASTX searching. More than 75% of the significant hits were the same as those observed in algae such as *Ectocarpus siliculosus* (54%) and *Nannochloropsis gaditana* (20%) (Fig. 2a).

To classify the function of the assembled transcripts, Gene Ontology (GO) assignment was carried out. In the “Biological Process” category, the top three GO terms were “metabolic process”, “cellular process”, and “single-organism process”. In the “Cellular Component” category, the top three GO terms were “cell”, “cell part”, and “organelle”. In the “Molecular Function” category, the top three GO terms were “catalytic activity”, “binding”, and “transporter activity” (Fig. 2b). To further analyze the transcript functions, KEGG pathway mapping was undertaken. The pathways were mainly involved in cellular processes, environmental information processing, genetic information processing, metabolism and organic systems.

### Difference in the expression of genes associated with carbon metabolism and lipid biosynthesis

Compared with photoautotrophic cells, there were 1032 over-expressed and 1442 down-regulated genes in heterotrophic cells. Among these genes, the differentially expressed genes (DEGs) associated with carbon metabolism and fatty acid and triacylglycerol biosynthesis were further analyzed (Table 2). We found that under heterotrophic condition, the expression of genes encoding phosphoenolpyruvate carboxylase (PEPC, which catalyzes the irreversible β-carboxylation of phosphoenolpyruvate to produce inorganic phosphate and oxaloacetate) and fructose-1,6-bisphosphatase (FBP, which catalyzes the formation of fructose-6-phosphate from fructose-1,6-bisphosphate and is a rate-limiting enzyme in carbon metabolism) was induced. This result indicated upregulation of gluconeogenesis pathway [42], which is accordant with the high levels of carbohydrate accumulation observed (Fig. 1). In contrast, we observed that the expression of most key genes involved in the glycolysis pathway was decreased under heterotrophic condition. These gene mainly include 6-phosphofructokinase 1 (PFK), glyceraldehyde 3-phosphate dehydrogenase (GADPH), phosphoglycerate kinase (PGK), enolase (ENO) and pyruvate kinase (PK). The downregulation of glycolytic enzymes under heterotrophic condition is inconsistent with the result of the study on *C. protothecoides* Cp0710 [36]. These results were unexpected, because decreased glycolysis could result in decreased accumulation of ATP and precursor like 3-phosphoglycerate, which are necessary for fatty acid synthesis [43]. Moreover, acetyl-CoA synthetase (ACS) and pyruvate dehydrogenase genes (PDH) are two important enzymes involved in the production of acetyl-CoA, which is the main precursor of fatty acid synthesis, via alternative routes. Genes encoding these two enzymes exhibited low transcript abundances under heterotrophic conditions, indicating the suppression of acetyl-CoA production. Interestingly, the levels of most enzymes involved in the tricarboxylic acid cycle (TCA cycle) were not significantly changed, but expression of the genes encoding malic dehydrogenase (MDH) which catalyzes the conversion of malate into oxaloacetate, was elevated under heterotrophic condition. Moreover, the gene encoding glutamate dehydrogenase (NAD(P)+) was also in high abundance. This finding may indicate that the accumulation of proteins and amino acids was activated under heterotrophic condition for maintenance of essential metabolic functions for rapid growth [44].

**Table 2:** Differentially expressed genes involved in central carbon metabolism and lipid metabolism in *T. minus* under photoautotrophic and heterotrophic conditions

Pathway	Gene symbol	Gene name	EC number	Fold change
Carbon metabolism	GAPDH	Glyceraldehyde 3-phosphate dehydrogenase	1.2.1.12	− 1.08
	PFK	6-Phosphofructokinase 1	2.7.1.11	− 3.22
	PK	Pyruvate kinase	2.7.1.40	− 1.25
	PDH	Pyruvate dehydrogenase E1 component alpha subunit	1.2.4.1	− 1.84
	FBP	Fructose-1,6-bisphosphatase	3.1.3.11	+ 3.82
	ENO	Enolase	4.2.1.11	− 1.75
	PGK	Phosphoglycerate kinase	2.7.2.3	− 1.67
	DLD	Dihydrolipoamide dehydrogenase	1.8.1.4	− 1.10
	PEPC	Phosphoenolpyruvate carboxylase	4.1.1.39	+ 2.89
	ACOS	Acetyl-CoA synthetase	6.2.1.1	− 1.53
	GDH	Glutamate dehydrogenase (NAD(P)+)	1.4.1.2	+ 3.27
	MDH	Malic dehydrogenase	1.1.1.37	+ 1.66
	TKT	Transketolase	2.2.1.1	− 1.65
	PGDH	Phosphogluconate dehydrogenase	1.1.1.343	− 1.41
Fatty acid biosynthesis	ACC	Acetyl-CoA carboxylase	6.4.1.2	− 1.24
	KASIII	3-Ketoacyl-CoA sythase III	2.3.1.180	− 1.14
	LACS	Long-chain acyl-CoA synthetase	6.2.1	− 1.29
	SAD	Stearoyl-ACP reductase	1.14.19.2	+ 1.24
	FAD	Acyl-lipid omega-6 desaturase	1.14.19	− 1.48
Triacylglycerol biosynthesis	GPAT	Glycerol-3-phosphate acyltransferase	2.3.1.15	− 0.35
	DGK	Diacylglycerol kinase	2.7.1.107	− 1.46

Biosynthesis of storage lipid in microalgae occurs in two steps involving fatty acid biosynthesis and triacylglycerol biosynthesis pathway. The first step of de novo fatty acid biosynthesis is the conversion of acetyl-CoA into manlonyl-ACP by acetyl-CoA carboxylase (ACC). In this study, the expression of the ACC gene annotated from the transcriptomic data was decreased under heterotrophic condition. In addition, KASIII is the first enzyme involved in the biosynthesis of C16:0-ACP (palmitoyl-ACP) from malonyl-ACP. Hence, the downregulation of the KASIII genes in *T. minus* cells under heterotrophic condition indicated suppression of C16:0-ACP production [45]. Once released in plastids, free fatty acids can be converted into acyl-CoA by long-chain acyl-CoA synthase (LACS). In this study, the expression of the genes encoding LACS was decreased under heterotrophic condition, indicating that the pathway was suppressed. Approximately, seven genes from the TAG biosynthetic pathway were identified in *T. minus* cells, and a majority of the genes did not exhibit significant changes under photoautotrophic and heterotrophic conditions. However, we observed that the expression of a gene encoding glycerol-3-phosphate acyltransferase (GPAT) was down-regulated under heterotrophic condition, which is in agreement with the previous findings in *Scenedesmus* species [46]. GPAT catalyzes the formation of phosphatidic acid from acyl-CoA, which is the first step in TAG biosynthesis, and suppression of GPAT resulted in decreased neutral lipid content in microalgal cells [47].

Hence, in conclusion, the results further revealed that the strongly up-regulated genes in heterotrophic cells were involved in gluconeogenesis and TCA cycle, while genes involved in glycolysis, pyruvate metabolism and fatty acid biosynthesis were down-regulated. Additionally, these data also revealed the importance of carbon precursor supplementation for increased lipid accumulation in *T. minus* under heterotrophic condition.

### Metabolomics analyses of *T. minus* under photoautotrophic and heterotrophic conditions

**Fig. 3:**
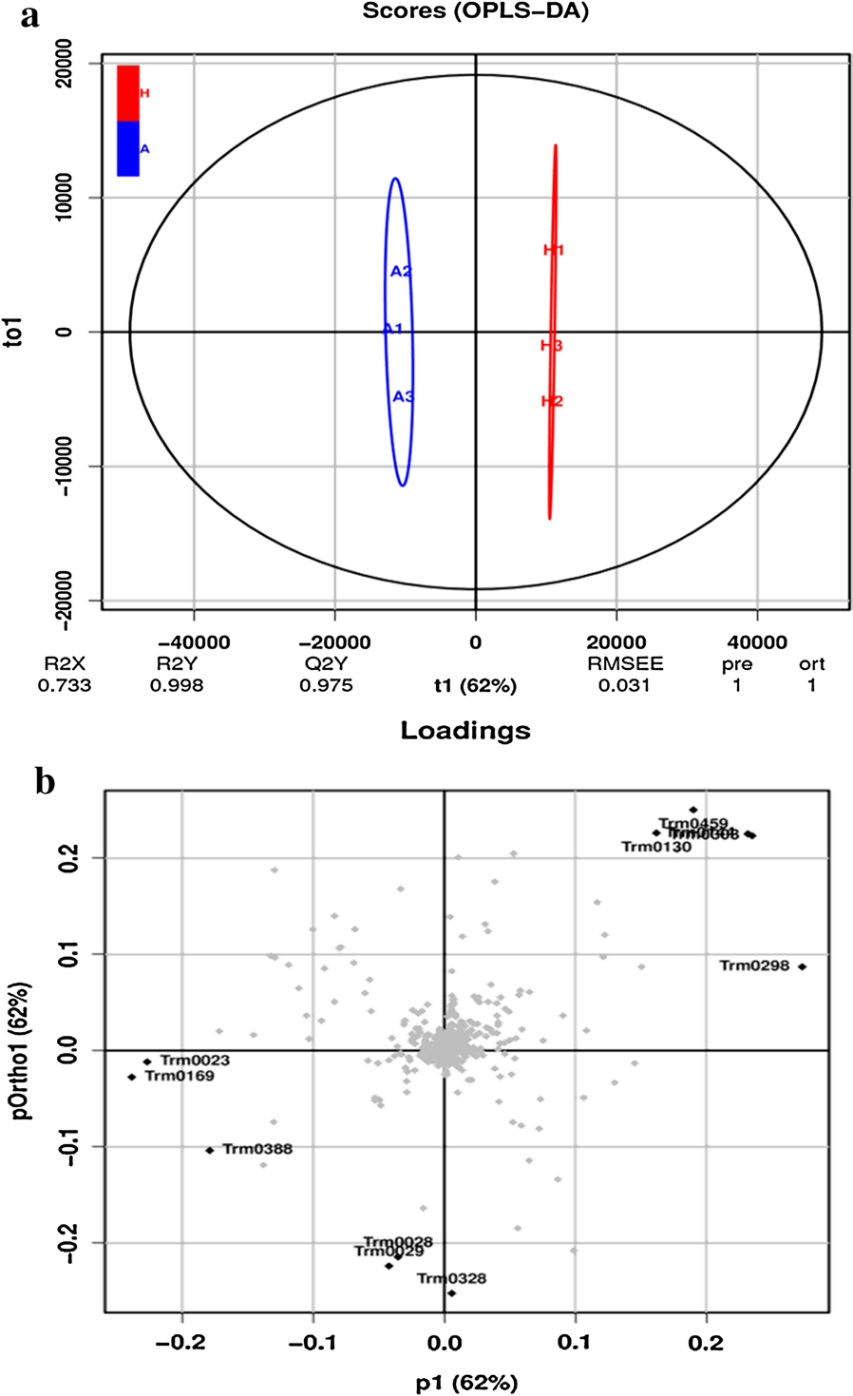
PCA analysis of metabolites in *T. minus* under photoautotrophic and heterotrophic conditions. **a** OPLS-DA score plot; **b** OPLS-DA loading plot with the parameters responsible for the culture conditions. A1, A2, A3: photoautotrophic samples; H1, H2, H3: heterotrophic samples

A total of 216 primary and secondary algal intracellular metabolites that were present in all samples were detected by LC–MS. Subsequently, we constructed traditional principal coordinate analysis (OPLS-DA) models to investigate the correlations between metabolite levels and culture conditions. The OPLS-DA model (Fig. 3a) represents the distribution of all data sets in the photoautotrophic and heterotrophic conditions. As shown in Fig. 3a, both models exhibited obvious clusters in the (*X*-axis) direction between the two groups, indicating that the heterotrophic culture was responsible for a majority of the differences between the metabolic profiles. Figure 3b showed the corresponding loading plots for the PLS-DA model to identify the metabolites responsible for the observed separation. As illustrated in Table 3, a total of 34 identified metabolites (13 amino acids, 2 sugars, 7 lipids, and 12 other substances) were significantly affected by the cultivation models.

**Table 3:** Overview of the differentially produced intracellular metabolites detected in *T. minus* under photoautotrophic and heterotrophic conditions

Class	Compounds	VIP	P value	log2_FC (heterotrophic/photoautotrophic)
Amino acid	l-Alanine	2.81	0.000189	2.69
	l-Proline	2.24	0.003812	0.96
	Phenylgycine	1.57	0.012838	0.71
	Homoserine	3.14	0.000475	1.29
	l-Histidine	2.41	0.00208	− 1.62
	l-Glutamine	4.91	8.58E−05	+ 1.62
	l-(−)-Tyrosine	3.88	0.004852	− 1.24
	l-(+)-Lysine	5.16	7.57E−05	− 1.74
	l-Phenylalanine	3.15	0.000252814	+ 0.35
	l-Tryptophan	3.88	0.002038562	− 1.23
	Citrate	2.49	0.0029875	− 0.85
	Succinate	3.27	0.00165524	− 1.06
	Trans-4-hydroxy-l-proline	1.09	0.001132	− 1.71
Alcohols	d-Mannitol	2.03	0.007543	− 1.41
	Dulcitol	3.72	0.002039	− 1.31
Carbohydrate	Fructose 6-phosphate	1.96	0.001094	1.34
	Glucose 6-phosphate	1.18	3.14E−05	1.15
Organic acid	dl-Pipecolinic acid	2.28	0.00292	− 1.47
	5-Aminolevulinate	1.27	0.000222	− 1.88
Lipid	Lyso PC16:0	1.64	0.01795	− 1.36
	LysoPC 16:2	2.81	0.04581	− 0.62
	Lyso PC 18:0	3.50	0.023243	− 1.66
	Lyso PC18:1	5.09	0.012256933	2.80
	Lyso PC18:2	5.91	0.001855198	− 1.67
	Lyso PS:20:0	4.11	0.025835	1.87
	Lyso PC:20:4	1.23	0.036285	− 2.29
Others	Phenolamides	1.08	0.005677	0.19
	Piperidine	1.28	0.010741	1.13
	*N*-Methyltyramine	1.13	0.025641	0.66
	Diosgenin	3.84	0.022532	0.62
	*N*-Acetylneuraminic acid	2.23	0.00182	− 1.72
	l-Methionine sulfone	2.99	0.027101	− 0.99
	2,6-Diethylaniline	1.00	0.001118	1.32
	*p*-Chlorophenoxyacetate	2.57	0.017218	− 2.78

The levels of 13 amino acids in *T. minus* were changed significantly under the two different culture conditions. Although we observed that the total protein content under heterotrophic condition was higher than that under photoautotrophic condition, some differentially produced amino acids still exhibited decreased abundances under heterotrophic condition. In detail, the levels of l-alanine, l-proline, homoserine, phenylglycine and l-phenylalanine were elevated, whereas the citrate and succinate levels were decreased, under heterotrophic cultivation. Citrate and succinate are two important participants in TCA cycle; moreover, citrate may be used as a carrier of acetyl units for fatty acid synthesis, and therefore, the reduction of citrate may partially explain the low levels of lipid accumulation, as discussed in previous study [48]. Significant reductions in the levels of l-histidine, l-(−)-tyrosine, l-(+)-lysine, l-tryptophan and trans-4-hydroxyl-l-proline were also observed in the heterotrophic cultures compared with the photoautotrophic cultures.

Based on metabolomic data, we identified 2 sugars and 2 alcohols that were exhibited differentially abundances between photoautotrophic and heterotrophic conditions. The sugars glucose 6-phosphate and fructose 6-phosphate, which are two important metabolites in the glycolysis/gluconeogenesis pathway, both increased dramatically. As mentioned above, the expression of fructose-1,6-bisphosphatease, which catalyzes the formation of fructose-6-phosphate from fructose-1,6-phosphate, was up-regulated under heterotrophic condition. Hence, the gluconeogenesis pathway might be elevated under heterotrophic condition, resulting in the high levels of glucose 6-phosphate and fructose 6-phosphate accumulation. In contrast, the abundances of mannitol and dulcitol were decreased in heterotrophic cultures. Mannitol and dulcitol provide carbon precursors via the formation of glucose 6-phosphate, leading to the synthesis of pyruvate in the glycolytic pathway for fatty acid biosynthesis. This result suggests that reduction in mannitol and dulcitol levels might lead to decreased lipid accumulation in *T. minus* cells under heterotrophic condition.

Meanwhile, the levels of seven fatty acids were also changed, and heterotrophic cultivation decreased the levels of 5 fatty acids in *T. minus*, namely, the Lyso PC 16:0, Lyso PC 16:2, Lyso PC 18:0, Lyso PC 18:2 and Lyso PC20:4. In plant cells, PC 16:0, 18:0 and 18:1 are the three common products of de novo fatty acid synthesis [49]. The decreased levels of these three fatty acids might lead to reduced lipid accumulation, which is consistent with our observation based on transcriptomic analysis.

In addition, stearoyl-ACP desaturase (SAD) synthesizes monounsaturated fatty acids from saturated fatty acids by introducing the first double bond [50]. As described above, the gene encoding the SAD enzyme was up-regulated under heterotrophic condition. Hence, the levels of the enzyme-catalyzed substances Lyso PC 18:1 were increased.

Besides amino acids, sugars and lipids, there were still 10 other differentially produced intracellular metabolites (Table 3) belonging to β-alanine metabolism, lysine metabolism, pyrimidine metabolism, and the alkaloid biosynthesis from terpenoid and polyketide. Among these metabolites, in plant cells, *N*-methyltyramine can be catalyzed by *N*-acetylneuraminicacid (Neu5Ac) to produce *N*-acetyl-d-mannosamine (ManNAc) and pyruvate, the latter is an important precursor in fatty acids biosynthesis. Therefore, the level of *N*-methyltyramine might influence the final lipid accumulation to a certain extent.

### Changes in the transcriptome and metabolome in response to heterotrophic condition

**Fig. 4:**
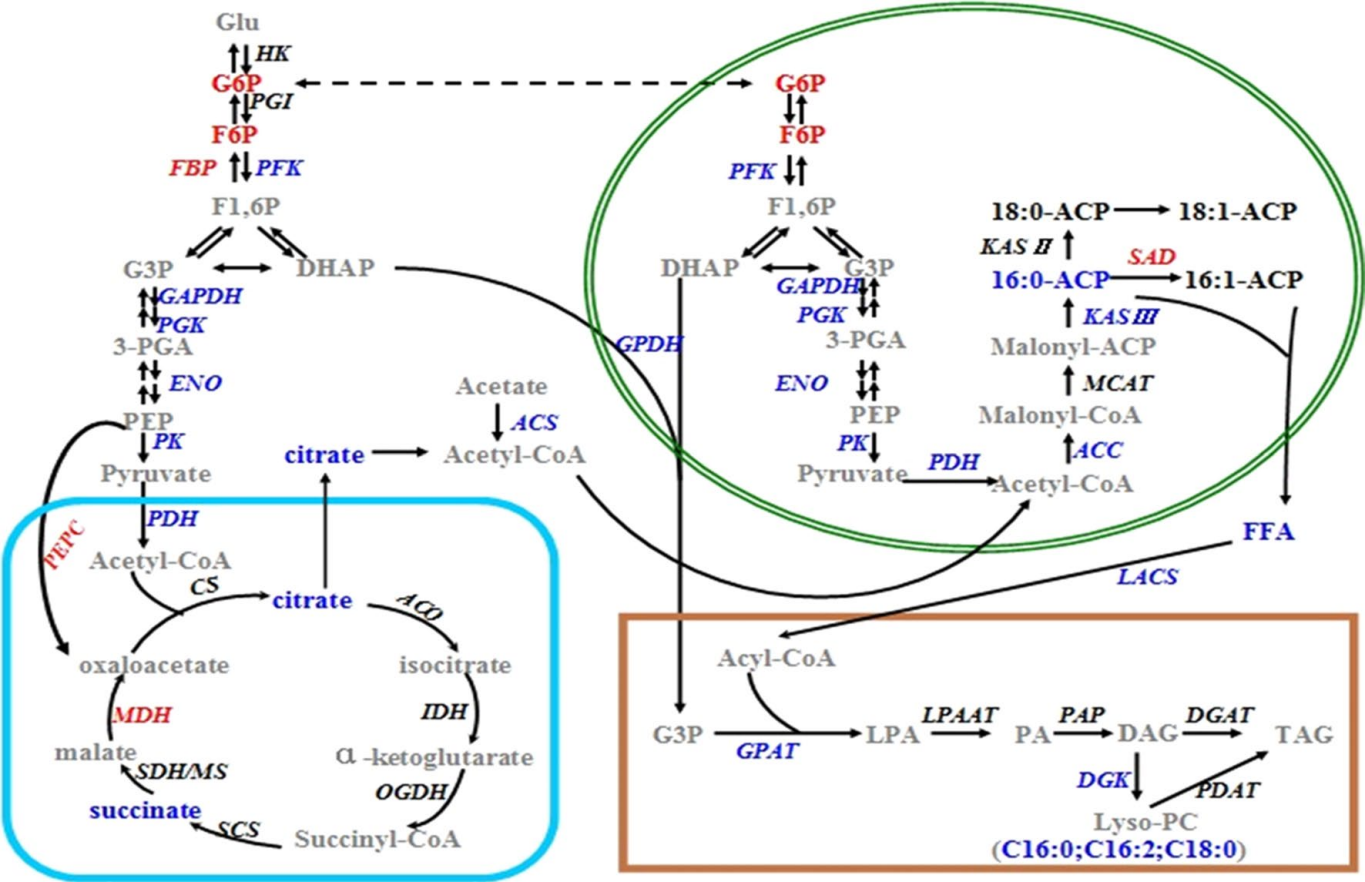
Regulatory model for the flow pathway of central carbon metabolism and a proposed scheme for lipid biosynthesis in *T. minus*. Transcriptomic genes and metabolites that exhibited increased abundances under heterotrophic condition are shown in red, those exhibit decreased abundances are shown in green, and those unchanged or undetected are in black (enzymes) or gray (metabolites) using both transcriptomics and Metabolomics profiles defined here

Based on transcriptomics, metabolite profiling and biochemical data, we proposed a simplified carbon precursor flow scheme for lipid biosynthesis under heterotrophic condition (Fig. 4). This scheme showed with reasonable clarity the flow of carbon precursors from glucose to the different metabolic pathways, mainly the glycolytic/gluconeogenesis pathway, TCA cycle and fatty acid biosynthesis. Briefly, most highly mapped enzymes involved in glycolytic, fatty acids and TAG biosynthesis were down-regulated, while the key enzyme involved in the gluconeogenesis was up-regulated under heterotrophic condition. Although most mapped metabolites under heterotrophic condition were not significantly different from those under photoautotrophic condition, the metabolomic data revealed that the levels of two key metabolites involved in glycolysis/gluconeogenesis were elevated, while several metabolites involved in the TCA cycle and fatty acid biosynthesis were present in low abundance.

In conclusion, the metabolomic data demonstrated that the carbon precursors generated via the glycolytic pathway were supplied largely to gluconeogenesis and TCA cycle, at the expense of fatty acids levels and TAG biosynthesis. The transcriptomics data set supported this interpretation by demonstrating that some genes encoding key proteins involved in glycolysis, acetyl-CoA synthesis, fatty acid biosynthesis and TAG synthesis were present at low abundance under heterotrophic condition. Furthermore, the metabolic map also revealed that the levels of the two carbon precursors (acetyl-CoA and glycerol-3-P) for lipid biosynthesis were limited under heterotrophic condition, which was explained by the decrease in the corresponding enzymes and C16:0 based on the transcriptomic and metabolomic data. Therefore, the lipid content was much lower under heterotrophic condition than that under photoautotrophic condition. Thus, the visualization and mining of enzymes and metabolite networks turned out to be a reasonably conservative approach for identification of biomarkers that might play a potential role in lipid accumulation. Among the metabolites that exhibited decreased levels under heterotrophic condition, the metabolites correlated with carbon precursors (citrate, succinate and l-glutamine) may contribute to lipid accumulation via fatty acid or complex lipid synthesis.

### Effect of potential metabolites supplementation on biomass and lipid accumulation under heterotrophic condition

**Fig. 5:**
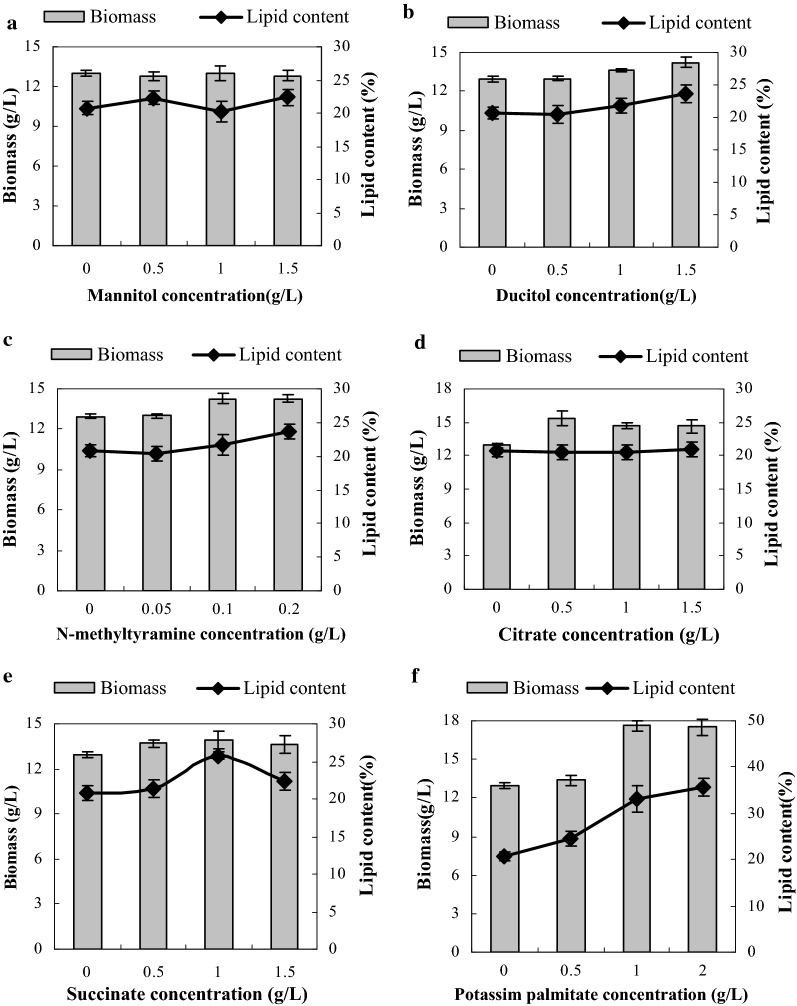
Effects of six potential metabolite markers on the biomass and lipid accumulation of *T. minus* under heterotrophic condition. **a** Mannitol; **b** dulcitol; **c** *N*-methyltyramine; **d** citrate; **e** succinate; **f** potassium palmitate

To determine whether metabolite markers would increase the accumulation of lipid in vivo as suggested by our transcriptomic and metabolomic analysis, six potential metabolites (dulcitol, mannitol, N-methyltyramine, citrate, succinate and potassium palmitate) were supplied at different concentrations. Figure 5a, b revealed the effects of supplementation with 2 sugars (mannitol and dulcitol) on the biomass and lipid accumulation of *T. minus* under heterotrophic condition. Mannitol supplementation did not cause a significant change in the growth and lipid accumulation of *T. minus* in heterotrophic cultures. Moreover, cultures supplemented with dulcitol achieved slightly higher final biomass and lipid content values than those that lacked additional dulcitol, although the differences were not statistically significant.

As indicated in Fig. 5c, the biomass and lipid accumulations of *T. minus* increased slightly as the concentration of *N*-methyltyramine increased, with final values of 14.3 g/L and 23.6%, respectively; The values were 13.0 g/L (biomass) and 20.8% (lipid content) in the absence of metabolite supplementation. Supplementation with citrate promoted the growth of microalgal cells, as shown in Fig. 5d, although the highest dry weight of 15.4 g/L appeared upon the addition of 0.5 g/L citrate. However, the lipid content of cultures supplemented with citrate was the same as that of control. This finding was different from the result of previous study [15], in which the addition of citrate stimulated fatty acid synthesis in *Chlamydomonas reinhardtii*. *T. minus* cultures supplemented with exogenous succinate, another important amino acid in TCA cycle (similar as citrate), exhibited some changes in both biomass and lipid accumulation in *T. minus* cells (Fig. 5e). With 0.5 g/L succinate supplementation, the biomass was 13.70 g/L with a slight increase from 12.97 g/L; however, at concentrations greater than 0.5 g/L, the biomass did not increase significantly. On the other hand, while the lipid content did not change upon supplementation with 0.5 g/L succinate, the value increased to 25.77% of dry weight with 1 g/L succinate; however, at concentrations higher than 1 g/L, the lipid content decreased to 22.4%.

Based on the transcriptomic data, most of the genes encoding enzymes involved in de novo fatty acid synthesis, including ACC, MAT, KASIII, KAR, HAD and EAR, were down-regulated or unchanged under heterotrophic condition. Hence, supplementation with palmitic acid might promote total lipid accumulation. Considering the weak solubility of palmitic acid, we used the soluble potassium palmitate instead of palmitic acid. As indicated in Fig. 5f, addition of potassium palmitate stimulated both the growth and lipid accumulation of *T. minus* cells. The highest biomass value increased significantly to 18.62 g/L, which was ca. 1.5-fold of control, upon addition of 1 g/L potassium palmitate. Meanwhile, upon addition of 0.5, 1 and 2 g/L potassium palmitate, the lipid levels achieved were 24.52, 32.98 and 35.59%, respectively, which were much higher than the initial value of 20.76% of dry weight observed for the control. Without doubt, potassium palmitate is an effective trigger that promotes intracellular lipid accumulation in heterotrophic cultures. Of course, the lipid content remained lower than the value of 55% observed in photoautotrophic cultures, optimization of various parameters, including dose and time of potassium palmitate supplementation, and combination with other strategies may further elevate lipid level under heterotrophic condition.

## Conclusions

Suppressed lipid accumulation in heterotrophically cultivated *T. minus* might result mainly from down-regulation of glycolysis, de novo fatty acids and lipid biosynthesis, and up-regulation of gluconeogenesis. Thus, the insufficient supply of carbon precursors caused low levels of lipid accumulation during heterotrophic cultivation. Supplementation with exogenous carbon metabolites, especially potassium palmitate, dramatically increased the total lipid content in *T. minus* grown in heterotrophic culture. The genetic regulation of metabolic targets in *T. minus* indicated by omics data will be studied further.
